# Characterization and printability of Sodium alginate -Gelatin hydrogel for bioprinting NSCLC co-culture

**DOI:** 10.1038/s41598-019-55034-9

**Published:** 2019-12-27

**Authors:** Arindam Mondal, Aragaw Gebeyehu, Mariza Miranda, Divya Bahadur, Nilkumar Patel, Subhramanian Ramakrishnan, Arun K. Rishi, Mandip Singh

**Affiliations:** 1 College of Pharmacy and Pharmaceutical Sciences, Florida A&M University, Tallahassee, Florida 32307 USA; 2 Department of Chemical & Biomedical Engineering, FAMU-FSU College of Engineering, Florida State University, Tallahassee, FL 32310 USA; 3 John D. Dingell VA Medical Center, Detroit, MI 48201 USA; 4 Department of Oncology, Karmanos Cancer Institute, Wayne State University School of Medicine, Detroit, MI 48201 USA

**Keywords:** Biomaterials - cells, Non-small-cell lung cancer

## Abstract

3D bioprinting improves orientation of *in vitro* tumor models by offering layer by layer positioning of cancer cells and cancer associated fibroblasts (CAFs) which can replicate tumor microenvironment. Aim of this study was to develop a sodium alginate -gelatin (SA-GL) hydrogel by optimizing rheological parameters to print non-small cell lung cancer (NSCLC) patient derived xenograft (PDX) cells and lung CAFs co-cultures. SA-GL hydrogels were prepared, and rheological properties were evaluated. Both the cells were mixed with the hydrogel and printed using INKREDIBLE bioprinter. Hydrogels prepared with 3.25% and 3.5% (w/v) SA and 4% (w/v) GL showed higher printability and cell viability. A significant decline in viscosity with shear rate was observed in these hydrogels suggesting the shear thinning property of hydrogels. Spheroid size distribution after 15 days was in the diameter range of 50–1100 µm. Up-regulation of vimentin, α-SMA and loss of E-cadherin in co-culture spheroids confirmed cellular crosstalk. This study demonstrates that rheological optimization of SA-GL hydrogel enhances printability and viability of NSCLC PDX and CAF co-culture which allows 3D co-culture spheroid formation within the printed scaffold. Therefore, this model can be used for studying high throughput drug screening and other pre-clinical applications.

## Introduction

Bioprinting technology is gaining widespread popularity in cell culture as it offers layer by layer positioning of different cells within 3D space. Extrusion bio printing allows high cell density printing with less process induced cell damage compared to other approaches. Biomaterials with a wide viscosity range can be precisely printed by extrusion bioprinting^1^. Different types of crosslinking process e.g. ionic, photo and thermal can be used during and after extrusion bioprinting^2^. Moreover, the shape and architecture of the scaffold can be defined using computer aided design (CAD) from clinical imaging data. Complexity of the biological tissues can be fabricated from MRI or CT scan images using multiple biomaterials and multiple print heads^3^. Intracellular interactions between cancer cells, cancer associated fibroblasts and cancer stem cells coordinate various cancer hallmarks like tumor heterogeneity, epithelial mesenchymal transition, invasion and migration^4,5^. Further, 3D bioprinting bridges between artificially engineered tissue-constructs with the native tissue by offering spatial distribution and recapitulation of personalized architectural accuracy^6,7^.

To print cancer cells, hydrogel plays a key role in high structural fidelity with enough cell viability. Moreover, hydrogels’ physical property has significant impact on cell proliferation, migration, aggregation and normal cell activities in the bio-printed scaffolds^8–11^. Various types of synthetic and natural polymers have been used for hydrogel preparation depending on their biocompatibility, water absorbing ability and gel strength^12^. Due to favorable proteolytic degradability, matrix stiffness and cell adhesion properties, synthetic biomaterials such as poly (ethylene glycol) (PEG), poly (n-isopropyl acryl amide) (pNIPAAm), and poly (caprolactone) (PCL), poly (D, L-lactide-co-glycolide) (PLGA) are also gaining popularity in 3D bioprinting research. Recent reports have shown that the natural biomaterials like alginate, chitosan, gelatin, collagen, and hyaluronic acid can also be used to build 3D cell laden scaffolds^6,13–18^. These natural polymers have good control over quantity of ECM proteins and growth factors. Recent studies by several investigators have used combination of sodium alginate(SA) and gelatin(GL) as a potential bioprintable hydrogel because of its cell compatibility and ability to form 3D spheroids^6^. SA is the common natural polymer for hydrogel preparation due to its potential biomedical applications and possibility of formulating wide range of hydrogel viscoelasticity by ionic crosslinking with CaCl_2_. Further, CaCl_2_ crosslinked SA was reported as biocompatible and does not affect cell viability during the gelling process with a concentration range up to 100 mM^19^.

GL has also been utilized in combination with SA as a hydrogel for printing tumor cells. GL at 37 °C is a solution but can form gel at <29 °C due to its conformational change from coil to helix. This property of GL is used to control the viscosity of alginate solution and thereby printable at different temperature range^20^. Pan *et al*. (2016), Li.Z *et al*. (2018), Chung *et al*. (2013), and Jiang *et al*. (2017) have already demonstrated the printability and biocompatibility of SA/GL hydrogel for printing of various living cells^8,21–23^. However, characterization of rheological properties e.g. viscosity, viscoelasticity, shear thinning, or shear thickening behaviors of hydrogel for bio-printing of patient specific cancer cells has not been well described. The presence of CaCl_2_ crosslinked SA matrixes and the coacervation between SA and GL, increases the thermal stability of GL and its delay in GL exudation from the bio fabricated scaffolds at 37 °C^24–26^.

Among advanced non-small-cell lung cancer (NSCLC) patients, epidermal growth factor receptor (EGFR) T790M mutation is the most common mechanism which results in approximately 60% of acquired resistance to first generation tyrosine kinase inhibitors (TKIs). Several preclinical studies with various treatment strategies have been reported but disappointingly acquired resistance to EGFR-TKIs develops within 12 months of treatment^27,28^. One of the reasons for the higher rate of clinical application failures in NSCLC drug discovery is currently available 2D models cannot replicate the three-dimensionality and heterogeneity of the tumor microenvironment. 3D cell culture can represent a stringent model for *in vitro* drug screening due to possessing several *in vivo* features such as cell-cell interactions, drug penetration, drug response, drug resistance and production of extracellular matrix^29,30^. Moreover, Patient derived tumor xenograft cells (PDX) in 3D culture recapitulate patient specific tumor microenvironment *in vitro* and can generate promising outcomes in preclinical drug evaluation. A major portion of non-small cell lung cancer (NSCLC) tumor stroma consist of Cancer Associated Fibroblasts (CAFs) which are associated with NSCLC progression, angiogenesis, epithelial to mesenchymal transition and metastasis. CAFs were also responsible for both intrinsic and acquired resistance to Tyrosine Kinase Inhibitor (TKIs) of NSCLC harboring T790M second mutation in EGFR^31^. Therefore, distinctive micro-environmental influences on NSCLC *in vitro* tumor model with co-cultured CAFs and patient derived tumor cells (EGFR T790M mutants) will be helpful for better understanding of stromal contribution to NSCLC. However, choice of materials and bioprinting of NSCLC PDX and CAFs co-culture with consistent viability and functionality currently are not well established.

The use of majority of reported natural or synthetic hydrogels as bioprinting material is limited due to biocompatibility and printability issues. Different hydrogels interact differently with different cell types; therefore, development of a hydrogel for printing cancer cells is very important. The primary requirements for a bioprinting material are a) Cell viability, b) printability (shear-thinning, compatible printing pressure, continuous extrusion without air bubbles), c) structure stability (stability of printed structure before crosslinking and after crosslinking), and d) non- toxic crosslinking. In this context we attempted to address all the important factors to develop a NSCLC co-culture platform using 3D bioprinting. This study for the first time aims to investigate a) Co-culture spheroid growth using patient derived NSCLC cell line and lung cancer associated fibroblasts (CAFs) in bioprinted scaffold. b) Printability of SA-GL hydrogels by tuning SA and GL concentration to improve cell viability and spheroid formation after bioprinting. c) a correlation between hydrogel rheology and patient derived cell survival and co-culture spheroid formation and d) scaffolds stiffness and stability during incubation period.

## Results

### Printability of SA and GL hydrogels

SA was used as a main component for bioprinting. SA solution at low concentration (<4%w/v) showed very low viscosity and ill-defined structural infusion between the spaces which made it difficult to print. In contrast, fully interconnected, well defined structures of scaffolds were printed after addition of GL to the SA solution. In the preliminary study, various hydrogels with 1–6% (w/v) SA and 3–8% GL were prepared and printed at room temperature using the INKREDIBLE bioprinter (Cellink, Sweden). Scaffolds with 3% SA with 4% GL (S1) showed deformation of the printed structure but 3.25–4% SA with 4% GL (S2) showed high structure fidelity. The extrusion pressures for printing 3, 3.25, 3.5, 3.75, 4% SA with 4% GL (named as S1, S2, S3, S4 and S5) at room temperature were approximately 20 kPa, 35 kPa, 60 kPa, 90 kPa and 120 kPa respectively. The extrusion pressure for printing hydrogels with 7:3, 6:4 and 4:8 (SA and GL ratios) was approximately 115 kPa, 145 kPa and 250 kPa respectively.

### Viscoelasticity of hydrogels

**Figure 1:**
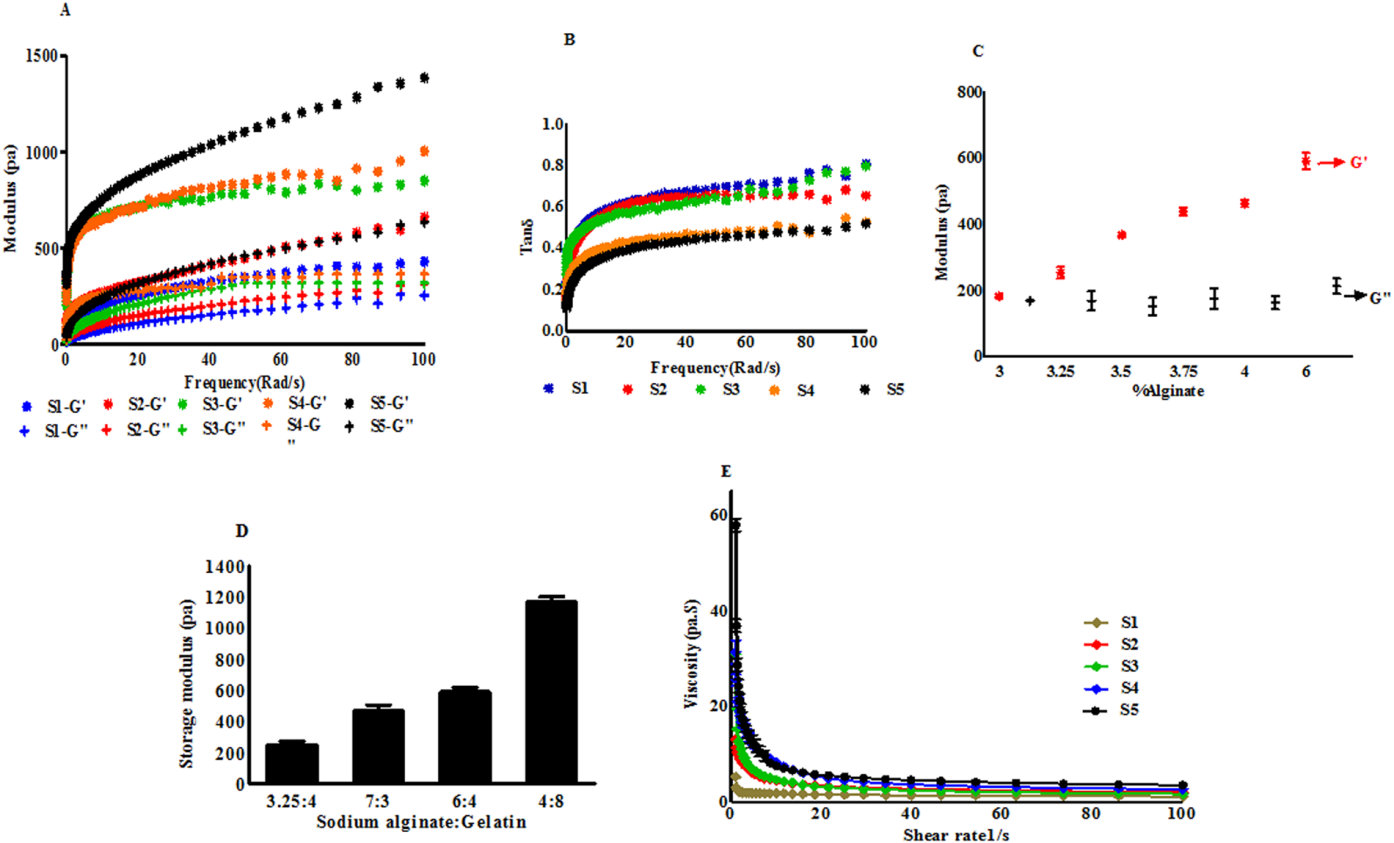
Rheological characterization of hydrogel mixtures with S1, S2, S3, S4 and S5 (3.0, 3.25, 3.5, 3.75 and 4.0% (w/v) SA and 4% (w/v) GL respectively). (**A**) Storage modulus G′ and loss modulus G″ under different angular frequencies. With the increase in SA concentration storage and loss modulus was increased. (**B**) Tan δ vs frequency of the hydrogel mixtures. Tan δ values were found <1 for all the hydrogel mixtures. (**C**) Storage modulus G′ and loss modulus G″ at different concentrations of SA from oscillation time sweep. Storage modus was increased with the increase in SA concentration. (**D**) Histogram shows the increase in storage modulus with increasing SA and GL concentrations in the hydrogel. (**E**) Viscosity of hydrogels at different shear rate. Viscosity of all the hydrogel mixtures decreased with increasing shear rate.

The rheological properties of five hydrogel samples with different concentrations of SA and fixed GL concentration were assessed. The viscoelasticity data of all sample hydrogels before and after crosslinking with CaCl_2_ showed higher storage modulus (G′) than loss modulus (G′′) at room temperature (25 °C). Also, with the increase in SA concentration, storage modulus was increased which suggested that the mechanical properties of the hydrogel increased. Figure 1(A) shows that at 0.1 Hz for S1, S2, S3, S4 and S5, the storage modulus was approximately 59.22, 104.42, 183.62, 272.28 and 371.75 respectively and the loss modulus was approximately 11.48, 16.25, 25.54, 34.70 and 42.52 respectively. Both storage and loss modulus were found to increase as the frequency increased. At 100 Hz the storage modulus for S1, S2, S3, S4 and S5 was approximately 429.08, 650.61, 994.75, 1666.74 and 1991.60 respectively and the loss modulus was approximately 389.89, 458.23, 547.72, 749.72 and 700.36 respectively. For all the hydrogels, tan δ values were calculated and are shown in Fig. 1(B) Tan δ vs frequency of the hydrogel mixtures. Tan δ values were found <1 for all the hydrogel mixtures. The strength of SA/GL hydrogel was increased as SA concentration increased in the hydrogel. Figure 1(C) demonstrates that increasing the concentration of SA in the system increases the elastic modulus of the system. Figure 1(D) also demonstrates the increase of storage modulus with increased concentration of both SA and GL. For this preliminary study, 3.25% SA: 4% GL was chosen as optimized printable hydrogel based on shape fidelity, storage modulus, cell viability and spheroid growth rate.

### Flow curve

The flow properties of non-crosslinked hydrogels were the main determinant factor for cell viability during printing process. At 25 °C, viscosity of all the hydrogels was decreased with increasing shear rate. Figure 1(E) shows that at low shear rate (1 s^−1^), the viscosity of the hydrogels was 4.33 ± 0.37 Pa.s, 12.10 ± 1.35 Pa.s, 31.96 ± 3.41 Pa.s, 26.57 ± 3.37 Pa.s and 55.47 ± 6.38 Pa.s for S1, S2, S3, S4 and S5 respectively. Again, at 100 s^−1^ the viscosity was decreased to 1.08 ± 0.92 Pa.s, 1.55 ± 0.21 Pa.s, 1.75 ± 0.29 Pa.s, 1.71 ± 0.53 Pa.s and 2.25 ± 0.31 Pa.s respectively for S1, S2, S3, S4 and S5. The decrease of hydrogel viscosity with the increase of the shear rate demonstrated the shear thinning property of the hydrogel.

### Bioprinted scaffold characterization

**Figure 2:**
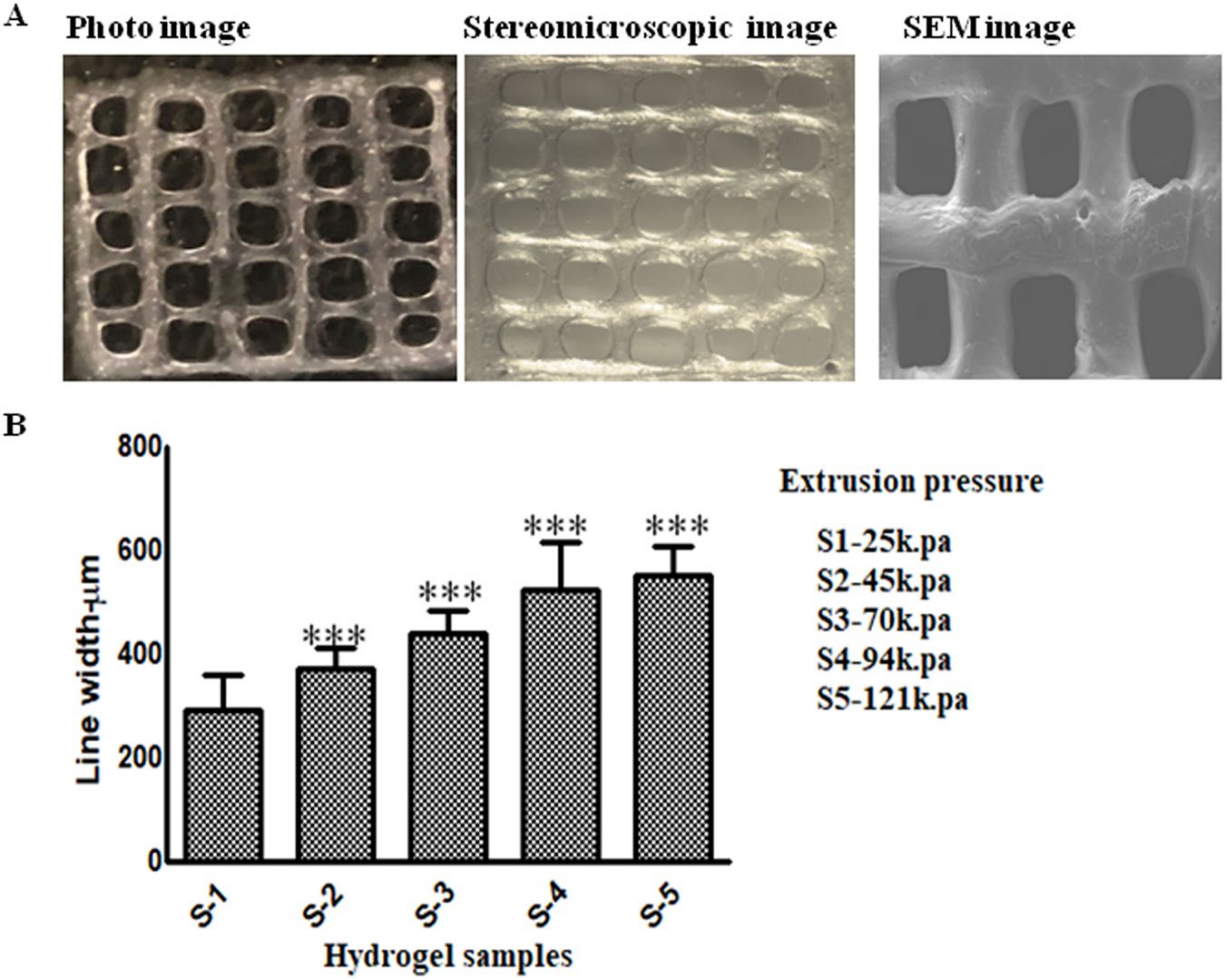
Characterization of scaffold printed with S-2. (**A**) Photographs taken with camera, stereomicroscope and SEM showed similar pore size with minimum line width. (**B**) Histogram showing line width measurement of bio-printed scaffold printed with S1, S2, S3, and S4 and S5. Increase in SA concentration in 4% GL increased the hydrogel modulus which requires higher extrusion pressure during printing. Higher pressure deposited more hydrogel and increase in the line width was observed.

For 3.25% SA and 4% GL, the photo image, stereomicroscopic micrograph and SEM showed similar pore size in the printed scaffold (Fig. 2A). Line widths of the printed scaffolds were found to be 293.09 ± 81.77 mm, 372.59 ± 39.38 mm, 441.59 ± 41.62 mm, 524.50 ± 91.31 mm and 552.72 ± 57.05 mm for S1, S2, S3, S4 and S5 respectively. Figure 2(B) shows that S1 had the smallest line width compared to S4 and S5. Line width for S2 and S3 was found approximately similar to the nozzle diameter. S1 was printed with 15–25 kPa extrusion pressure whereas S5 was printed at 100–125 kPa pressure. S5 has the biggest pore size with intermittent line width with lowest cell viability compared to the other samples.

### Bioprinted scaffold stiffness and stability during incubation period

**Figure 3:**
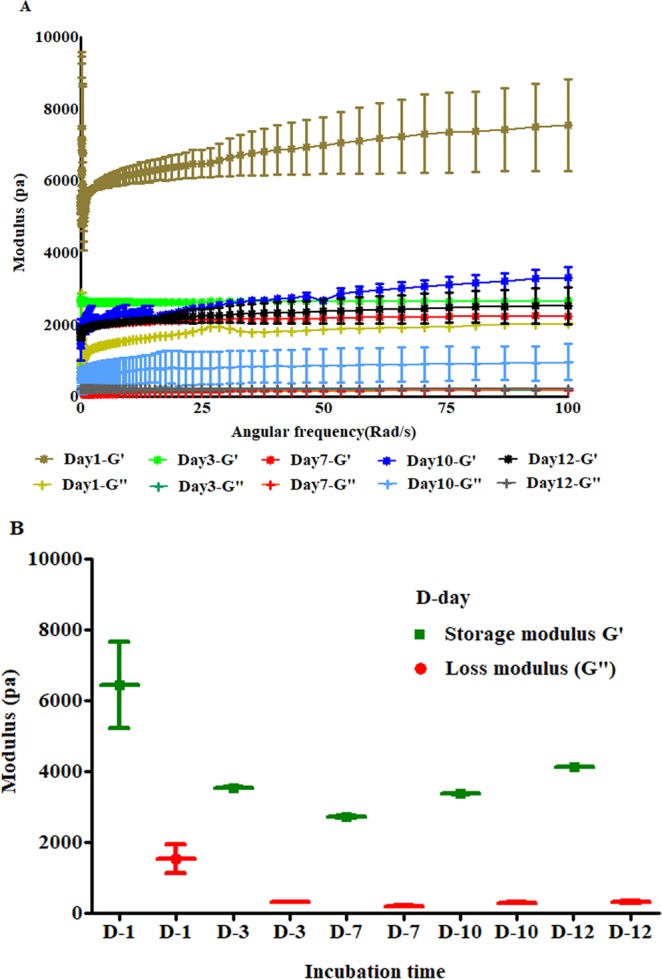
Stiffness of printed scaffolds evaluated in wet conditions. (**A**) Stiffness of cell laden scaffolds on 1, 3,7, 10 and 12 days post-incubation. (**B**) Stiffness of cell laden scaffolds measured with oscillation time test modes.

The stiffness of printed cell laden scaffolds at day 1,3,7,10 and 12 is depicted in Fig. 3(A,B) was evaluated by oscillation-frequency sweep and oscillation-time sweep measurement mode respectively. In all measurement, the storage modulus was higher than loss modulus and parallel in all frequencies (Fig. 3A). At day 1, scaffolds had higher stiffness which gradually decreased during the incubation period. The stiffness of all cell laden scaffolds was in the range of 1 kPa-8 kPa for 12 days. Figure 3(B) shows the stiffness of cell laden scaffolds which were evaluated by oscillation time sweep mode.

### Viability of printed PDX cells

**Figure 4:**
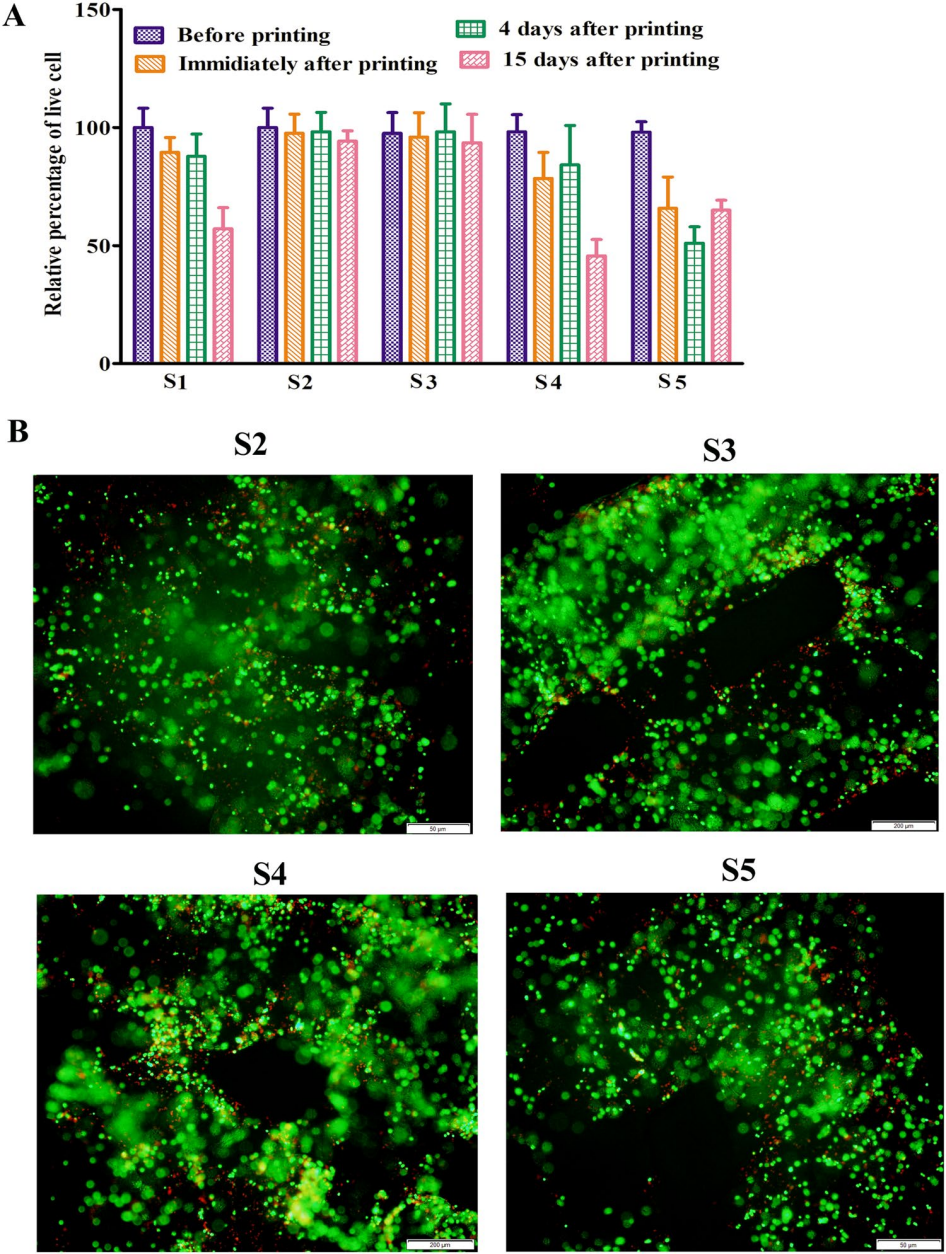
The viability of bio-printed PDX cells. (**A**) Histogram shows the viability of PDX cells before printing, immediately after printing, 4 and 15 days after bioprinting with S1, S2, S3, S4 and S5 hydrogel. The viability of the cells was higher before and immediately after the bioprinting in all the hydrogels. After 4 days, the cell viability was higher in S1, S2 and S3 hydrogel than S4 and S5 hydrogel. After 15 days, more than 50% cells were found dead in both S4 and S5 hydrogel but S2and S3 showed highest cell viability. (**B**) Live-DEAD staining of printed PDX cells 1 day after bioprinting. Representative images show live cells (green) and dead (red) cells.

Live-dead assay result (Fig. 4A) shows the percentage of viable PDX cells immediately after printing and were 89.47 ± 6.26, 97.51 ± 8.77, 95.98 ± 10.23, 78.44 ± 11.22 and 65.79 ± 13.24 for S1, S2, S3, S4 and S5 respectively. After 4 days, 87.77 ± 9.5, 98.23 ± 8.15, and 98.23 ± 11.74, 84.17 ± 16.69, and 51.05 ± 6.91 percent and after 15 days, 57.13 ± 8.91, 94.23 ± 4.33, 93.55 ± 12.04, 65.66 ± 14.09 and 45.65 ± 4.16 percent viable cells were observed in S1, S2, S3, S4 and S5 respectively. Figure 4(B) shows live (green) and dead (red) cells in the printed scaffolds 1 day after the bioprinting. Table 1 shows that hydrogels with 7:3, 6:4 and 4:8 SA and GL ratio results in approximately 71%, 63% and 52% cell viability respectively immediately after bioprinting.

**Table 1:** Correlation of extrusion pressure and cell viability.

Sodium alginate gelatin composition (%w/v)	3.25:4	7:3	6:4	4:8
Printing pressure (kPa)	45	115	145	250
Cell viability immediately after printing (approx.)	92%	71%	63%	52%

### Development of Co-culture spheroids within printed scaffold

**Figure 5:**
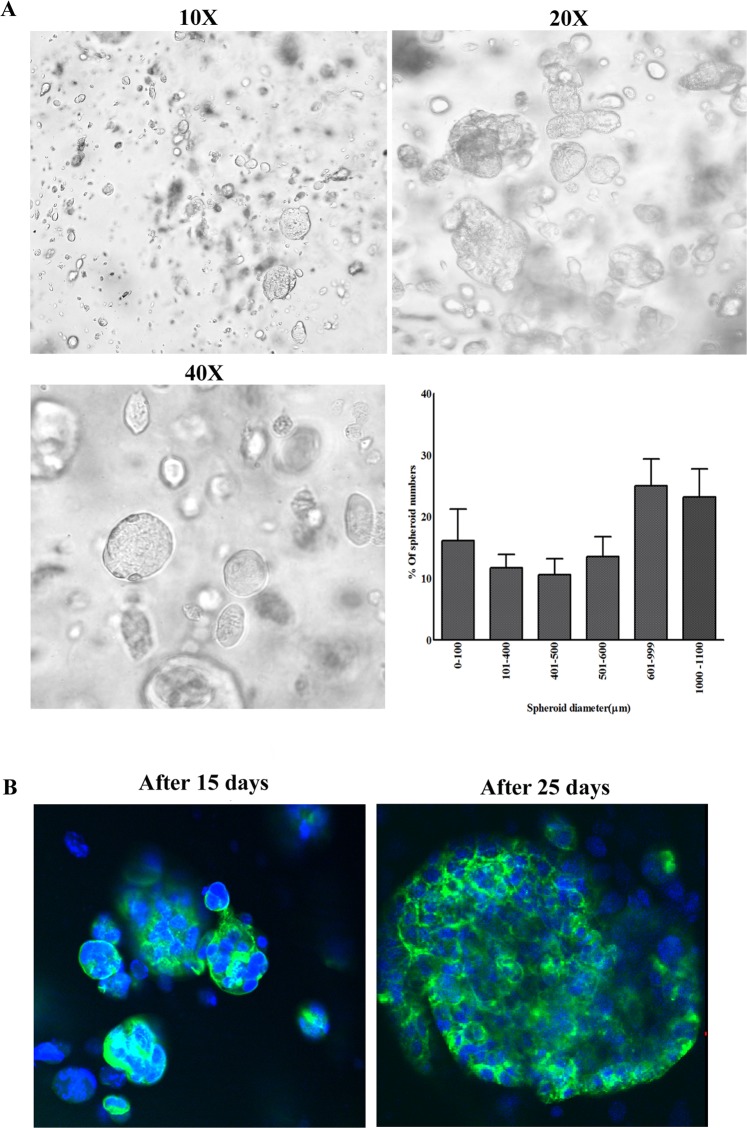
Bio-printed PDX-CAF co-culture spheroid formation. (**A**) Representative images show co-culture spheroid formation (10X, 20X and 40X magnifications) in the bio-printed scaffold using S2 hydrogel. Histogram shows the distribution of co-culture spheroids by size in the bio-printed scaffold. (**B**) Cytoskeleton distribution of co-culture spheroids in printed scaffolds after staining with actin (green) and nucleus (blue) on day 15 and 25. Increase in co-culture spheroid size with time was observed.

PDX and CAFs were observed to form small spheroids within the cell laden construct after 4 days of bioprinting. The spheroid size was increased over time. After 15 days, the spheroid size distribution pattern was characterized. Figure 5A shows spheroid size distribution in the printed scaffold and the sizes were in diameter range of 50 µm–1100 µm. At day 15, 25% of the spheroids had a spheroid size diameter range of 600 µm–1000 µm. NucBlue-ActinGreen staining in Fig. 5B shows the cytoskeleton of the printed co-culture spheroids on days 15 and 25.

### Crosstalk between PDX and CAF

**Figure 6:**
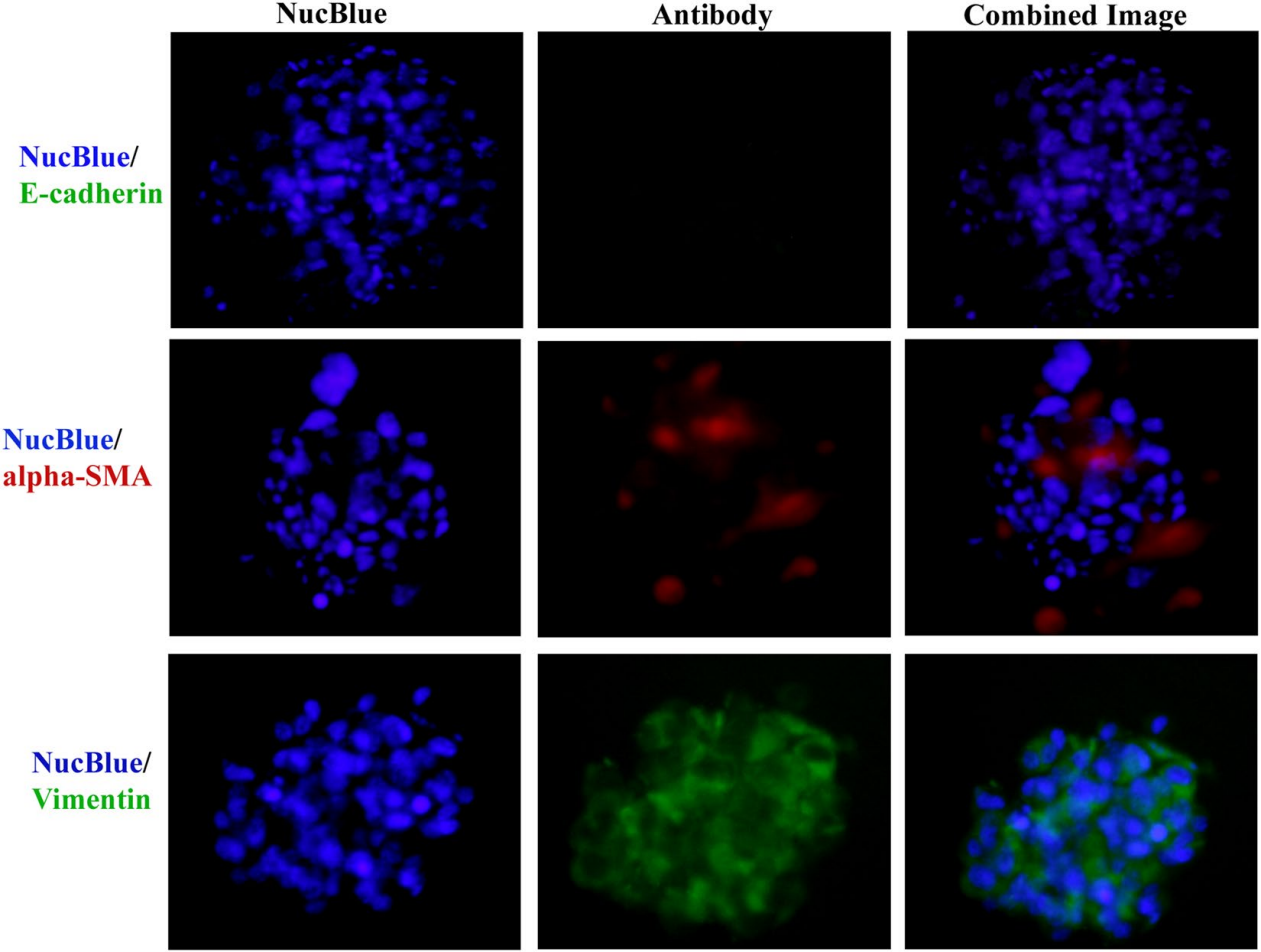
Immunostaining for co-culture spheroids for E-cadherin, vimentin and alpha-SMA. Co-culture spheroids were fixed, permeabilized and immune-stained for anti-E-cadherin (Green), anti-alpha-SMA (red), anti-vimentin (green) and Nucblue (blue) for nucleus. (Scale bar: 50 μm). Immunofluorescence staining showed higher expression of alpha-SMA and vimentin and down regulation of E-cadherin.

Figure 6 shows the loss of the cell adhesion molecule E-cadherin in the printed co-culture cells. The higher expressions of mesenchymal marker vimentin was also observed. CAFs in the printed cells showed higher expression of α-SMA levels. This phenomenon was  observed in various cells and spheroids.

## Discussion

Despite significant improvement in both technology and scientific approaches, NSCLC remains a leading cause of cancer associated deaths worldwide. Development of drug resistance and mutations in particular genes has decreased the treatment outcome as well as survival of the patient^32,33^. NSCLC PDX cells retain most of the mutations, pathologic and molecular characteristics of the original tumor. Moreover, PDX models are considered valuable in preclinical trials and the results will be used as a therapeutic guideline for future patients^34^. CAFs are important in the tumor microenvironment as they intensify tumor proliferation, angiogenesis, invasion and metastasis. CAFs help in TME remodeling and facilitate CAF activation and maintenance. Activation of CAFs results in cancer progression. CAFs are also known as one of the main regulators of tumor architecture and ECM. The synthesis and degradation of tumor architecture and ECM involves collagen, fibronectin secretion, and matrix metalloproteinase (MMP). CAFs are also considered as one of the most promising targets because genetically CAFs are normal, do not vary among patients and most importantly are less prone to evolve resistance^35–37^. Addition of CAFs with PDX cells into the 3D tumor model improves predictability of that model as well as enables better understanding and predictability of patients TME^38,39^. Although many co-culture models with 3D structures were reported with CAFs and tumor cells, most of them fail to provide a better understanding of the organization of different cells in the tumor microenvironment. Our hypothesis for this experiment was to develop an *in vitro* CAFs and tumor cells containing co-culture model which will enable studies to understand CAF-tumor cell interaction as in cancer patients. Further, this is the first report where we have used a SA-GL scaffold, which has been optimized using rheological parameters, to print tumor cells and CAFs using a 3D bioprinter which has shown to form viable spheroids.

Bioprinting technology not only allows/augments the cells to form spheroids but also facilitates the spheroids to form in different layers of the scaffold to mimic *in vivo* tumor microstructure. Therefore, this study is important to precisely develop multicellular spheroids using  3D bioprinting technology. This study will also allow researchers to study the complexity of the disease and to advance preclinical drug screening in the future. Three fundamental properties for cell bioprinting, (i) formation of consistent and cylindrical fibers (ii) stack printed layers into a coherent structure without spreading and (iii) lower extrusion pressure to achieve minimum stress, were used during preliminary screening of various SA/GL mixtures for 3D bioprinting. From these observations we chose five SA-GL ratios for potential hydrogel material for 3D bioprinting. All the five hydrogels were printed with the same protocol to evaluate changes in shape and size of printed structure after crosslinking.

Geometrics have been considered as a principal element in the role of the 3D printed cell laden scaffolds biofabrication and the right lattice design will help to mimic cellular microenvironment in addition to the bio-ink physicochemical properties^40,41^. Complexity of the biological tissues can be fabricated using multiple biomaterials and print heads. In bio-printing, printed scaffolds have an interconnected pore structure which ensures nutrient diffusion within the construct. Moreover, this interconnected porous structure also allows waste products diffusion from the scaffold. Scaffold size and the pore size are important as the cells primarily interact within the scaffold. Using that information, scaffolds can be designed to study drug delivery mechanisms and high throughput drug screening in pharmaceutical development.

In extrusion bioprinting, mechanical properties of the hydrogel plays an important role to determine the printed structure accuracy and cell viability^15^. In this study, all the rheology data was measured and stored within the rheometer control software with relevant rheological parameters (i.e., shear rate, shear stress, torque, normal forces, and angular frequencies). Further, this study does not include any conductivity test since our main focus was on the rheological properties of the hydrogels. Hydrogels which have higher viscosity, experience high shear forces to extrude through the nozzle during printing. Shear thinning behavior enables highly viscous hydrogels printable with structure accuracy^15,42,43^. High extrusion pressures are required in highly viscous hydrogels due to nozzle jamming. On the flip side, spreading of hydrogels is also a significant issue to print low viscous hydrogels^44,45^. During extrusion, hydrogels provide mechanical protection to the cells which can maximize the cell viability. Highly viscous hydrogels result in lower cell viability due to high extrusion pressure which imparts higher shear stress to cells^8,17,44,46–48^. Giuseppe *et al*. (2018) showed that 5% Alg-6% GL hydrogels with lowest viscosity and mechanical strength showed highest MSC viability after printing^48^. Increase in storage and loss modulus with increasing concentration of its constituents suggests the strength of the hydrogel. In only alginate solution, G′′ is dominant over G′ and behaves as a viscous fluid in all frequencies. Dominance of G′′ indicates the fluid-like property of the material. Due to insufficient energy or storage modulus, SA solution could not hold the printed structure^49,50^. Chung *et al*. (2013) reported that above 25 °C GL hydrogel alone and GL with 2% SA behaves like fluid (G′′ > G′) and after cooling G′ increased rapidly and crossed over G′′ and showed gel like characteristics. This report also suggested that SA-GL hydrogel should be printed at lower temperature where the hydrogel exhibits gel-like behavior^51^. In our experiment, frequency oscillation data showed that increase in storage (G′) and loss (G′′) modulus for 3–4% SA-4% GL hydrogel mixtures. The Tan δ value below 1 suggested that all the hydrogel mixtures are more gel like mixture than liquid like. The viscosity curves of all the hydrogel mixtures showed a similar pattern which suggests that all the hydrogels had shear thinning behaviors and the viscosity of these hydrogels were dependent on the dry content of the hydrogel and temperature besides to shear force and shear rate.

It has also been observed that 4% GL contributes a profound viscoelastic property when mixed with 3–4% SA which resulted in consistent extrusion through the nozzle. Non crosslinked 3–4% (w/v) SA solution alone cannot form a stable structure upon printing. Addition of GL imparts structural integrity to the printed structure as GL physically crosslinks at the printing temperature. Previous reports have shown that GL provides elastic characteristics of the hydrogel and improves cell adhesion within the hydrogel and SA contributes to the viscosity of the hydrogel and scaffolds stiffness^22,47,52^. Also, with the increase in SA concentration, pore diameter increases, and filament width decreases due to increase in the viscosity and storage modulus of the hydrogel^23^. Here, we fixed GL concentration and varied printability of the hydrogel by changing SA concentration. With increasing SA concentration, the modulus values increased which indicates the gelling behavior of the hydrogel. Moreover, printed scaffold resolution is dependent on the material rheological property and bioprinter condition which includes nozzle diameter, print-bed temperature  and printing speed. The scaffold width depends on the inner diameter of the printing nozzle and the viscoelastic property of the hydrogel. The small nozzle diameter with high extrusion pressure results higher resolution but it increases cellular damage. Previous studies reported that higher nozzle diameter with low pressure printing results a structure with low shape fidelity or limited resolution^53^. Here, SA-GL hydrogel with multiple process parameters was printed with 410 µm (inner diameter) nozzles and was found favorable for cell viability after printing.

Earlier studies conducted by other investigators established the printability and biocompatibility of SA-GL hydrogel with SA concentration 3–7 w/v% and GL concentration 6–8 w/v%^8,21,23,54^. In our experiments, all the hydrogels with 3.25–4% SA and 4% GL allowed construction of 3D scaffolds with good processability and shapes at room temperature. SA lower than 3.25% (w/v) and with 4% (w/v) GL could not hold the printed structure due to lack of required storage modulus. Giuseppe *et al*. (2018) reported that increasing the concentration of SA led to more accurate printing and higher concentration of GL (10%) reduced the printing accuracy^48^. In our experiments, 3.25% and 3.5% SA with 4% GL showed suitable flow properties through the nozzle tip at room temperature with superior shape fidelity and high cell viability immediately after the extrusion and 15 days after printing. Our results are supported by a study done by Katja *et al*. (2016) who showed that bioinks with viscosity range of 30-6 × 10^7^ mPa can be printable by extrusion type printer^8^.

Scaffold’s stiffness is also a vital parameter to maintain the structural integrity as well as tumor cell growth. Haobo *et al*. (2018) demonstrated that manipulation of mechanical stiffness of bio-fabricated 3D scaffolds helps to ensure customized physiological stiffness and mechanical support for high cell attachments. Further, stiffness of the hydrogel influences the cell proliferation and tumor spheroid growth^24^. Wang *et al*. (2014) and Yunfeng *et al*. (2018), reported that stiff hydrogel decreases cell proliferation by delaying/arresting cell cycle progression. Stiffer hydrogels create compact micro-environments which decrease cell growth and proliferation and our study suggests that the stiffness of hydrogels can be controlled by polymer concentration^55,56^. Cavo *et al*. (2016) reported that soft hydrogel allows cells to form bigger spheroids with time whereas the cluster size did not change^57^. In this study, we observed that the stiffness of printed cell laden scaffolds were in the range of physiological lung tumor stiffness which is reported to be in the range of 1 kPa to 50 kPa^58,59^. The stiffness of our bio-printed cell laden scaffolds was 1.00–8.00 kPa which is in the range of lung tumors stiffness.

In patient tumors, cancer cells may activate the stromal fibroblast into CAFs through stimulation of paracrine growth factor^60^. Transforming growth factor-β (TGFβ) helps to activate fibroblasts and platelet-derived growth factor (PDGF) from cancer cells play important role in fibroblast activation and proliferation^61,62^. Therefore, a mutual interaction between cancer cell induced fibroblast activation and fibroblast induced cancer proliferation and metastasis makes CAFs tumor supporter. In this study our main goal was to develop a method for co-culturing spheroids using bioprinting technology. We tried different ratios of PDX and CAFs for bioprinting (data not shown) but observed that at lower density, enough fibroblasts did not survive in the co-culture. As a preliminary study, we used a 50-50 cell density to develop the model. Further studies are required to optimize the hydrogel recipe (Bioink) which can enhance fibroblast growth. PDX and CAFs started forming spheroids within the printed heterogeneous cell laden scaffold 4 days after printing. Increasing spheroid size over time up to 15 days indicates proliferation of individual cells. Jiang et. al (2017) showed that bio-printed MDA-MB-231 and IMR-90 cells self-aggregated to form multicellular spheroids and the size of the spheroids increased over time^20^. In our study we found a similar trend of increasing spheroid size over time.

Current *in vitro* 3D cell culture models consist of mainly cancer cells whereas co-culture of cancer cell with CAFs is always required as CAFs are involved in the initiation and progression of the tumor. CAFs have been reported to induce Epithelial Mesenchymal Transition (EMT)^2,36,63,64^. Giannoni *et al*. (2010) showed the effect of human prostate CAFs in enhanced tumor growth, metastases and EMT in prostate cancer PC3 cells^65^. Soon *et al*. (2013) and Lebret et. al (2007) showed that CAFs induce EMT and influence resistance in human breast cancer MCF7 and PMC42-LA cell lines respectively^66,67^. Amann *et al*. (2014) demonstrated that co-culture of lung cancer A549 and Colo699 cells with SV80 fibroblasts induce EMT by modulating E-cadherin, vimentin, α-SMA and cytokeratin^68^. Further, Choe *et al*. (2015) showed that cross-talk between lung cancer PC9 and H358 cells and CAFs is responsible for EGFR-TKI Erlotinib resistance in NSCLC^69^. Although several studies mentioned a clear link between TKI resistance and EMT but there are very few effective NSCLC-CAFs co-culture models which have been developed for high throughput drug screening. This is the first report to develop a Lung T790M PDX cells co-cultured with NSCLC CAFs using 3D bioprinting technology. Here E-cadherin, was chosen as a marker for adherent cells, vimentin indicated the shift of epithelial cells into mesenchymal phenotype and α-SMA indicated mesenchymal phenotype of CAFs^68^. Moreover, expression of α-SMA typically identifies the presence of CAFs in the co-culture. In this study, down-regulation in E-cadherin, concomitant up-regulation of vimentin and higher expression of α-SMA in the printed co-culture spheroids suggested the crosstalk between CAFs and PDX cells which promotes EMT. Forming spheroids using patient derived cells with controlled and uniform size is always a challenge in cancer research. However, Cavo et. al. 2016 showed that the stiffness of the hydrogel determines the spheroid size^57^. Using rheological principles, other polymers can be used which may reflect similar cellular behavior and spheroid growth. Moreover, custom built bioprinting system may enable to generate a controlled and uniform spheroid by eliminating manual cell-seeding and non-uniform cell distribution in the hydrogel.

## Conclusion

We demonstrate that 3D bioprinting technology can be used to develop *in vitro* tumor co-culture spheroids by using optimized SA and GL hydrogel. This model also allows investigating tumor stroma interactions to mimic *in vivo* tumor microenvironments. However more detailed studies are required to overcome some other concerns, but this study preliminarily showed that increased cell viability and tumor-stroma interaction can be achieved in NSCLC PDX cells and CAFs co-culture through 3D bioprinting.

## Materials and Methods

Sodium alginate (MW 50000 Da) and gelatin type B from bovine skin (MW ~50,000–100,000 Da), calcium chloride analytical grade, DMEM/F12, RPMI, bFGF and EGF were purchased from Sigma-Aldrich (USA). B27 supplement, NucBlue, Actin-Green488 reagents were purchased from Life technologies (USA). Live-Dead assay reagent was purchased from Biotium Inc. (USA). Vimentin, E-cadherin and α-SMA were purchased from Cell Signaling Technology (USA). Anti-rabbit and anti-mouse FITC and Rh conjugated secondary antibodies were purchased from Santa Cruz biotechnology (USA).

### Preparation of hydrogels

GL type B was dissolved in 0.45%w/v saline solution at 40 °C for 2 h using overhead stirrer (LIGHTNIN, USA) and magnetic stirrer. SA powder was measured and mixed into the GL solution for 3 hours. The hydrogels were placed under UV light for 30 minutes and stored at 4 °C until use. Hydrogels were kept at room temperature for 3 h before printing.

### Rheological characterization of hydrogels

The mechanical properties of the hydrogels were studied using a combination of rotational and oscillatory rheology experiments on the AR 1500Ex Rheometer (TA instrument, USA). To load hydrogel sample, 2 ml hydrogel was placed on the lower plate of the rheometer and then melted to 34 °C for 3 minutes. The gap was set to 1 mm (parallel plate geometry, 20 mm diameter), and then cooled to 4 °C at a rate of 1 °C/min. Excess sample around trim gap was removed with spatula. For each measurement fresh samples were used with independent three replicates. The elastic and viscous moduli of hydrogels were evaluated at 25 °C using oscillatory rheology measurements (oscillation time sweep geometry) at a strain of 1% and frequency 10 rads^−1^. Next, frequency sweep measurements were carried out in the range 0.1–100 rad/s at a constant strain of 0.1% to show samples physical (viscoelastic and gel like) behaviors. Viscosities of all the samples were evaluated at 25 °C and at shear rate 1 to 100 s^−1^.

### Scaffold fabrication and hydrogel printability

Inkredible 3D bioprinter (CELLINK Inc, Sweden) was used to print all the hydrogels. The hydrogels were loaded into 3 ml printing cartridges (CELLINK Inc, Sweden) and connected to a controllable pressure regulator (0–1000 kPa). The hydrogel printing flow rate was optimized by adjusting the extrusion pressure (25–125 kPa) and Z-axis of 0.1 mm from nozzle tip to print bed. Before printing, hydrogels were taken out from the refrigerator and kept at room temperature for 3 h. Three layered scaffolds (10 × 10 × 1 mm) were printed on a petridish using 22 G nozzle (410 µm diameter). The dimensions of 3D printed scaffolds were measured after CaCl_2_ cross-linking. To measure the line width, scaffolds were printed (n = 5) with all the hydrogel mixtures. The line width of all the scaffolds was measured using CellSens software (Olympus, Japan).

### Bioprinted scaffold stiffness and stability during incubation period

The stiffness of scaffolds was evaluated with AR 1500Ex Rheometer (TA instrument, USA) with 1 mm measurement gap (parallel plate geometry, 20 mm plate diameter). To measure the stiffness of cell laden scaffolds, designed scaffolds with a 20 mm diameter,1 mm height and 65% infill density were printed and cross-linked with 100 mM CaCl_2_ for 3 minutes. Excess cross-linkers were removed and washed with HBSS. The scaffolds were incubated in 6 well plates with DMEM high glucose medium, at 37 °C with 5% CO2. The cultured scaffold was taken out from the well plates and viscoelasticity was ascertained at rheometer temperature of 37 °C and with plate to plate gap of 1 mm. Oscillation frequency sweep was run from 0.1 rad/s to 100 rad/s with 1% strain to check the frequency dependency stiffness of the scaffolds. Additionally, oscillation time sweep with a strain of 1% and angular frequency of 10 rad/s was used for stiffness measurement. The stiffness of the scaffold was measured at day 1, 3,7,10 and 12 at 37 °C. All scaffolds were constructed from 3.25% SA and 4% GL and each cell-laden scaffold had the initial estimated cells of 2 × 10^5^ cells per scaffold.

### Cell printing

NSCLC PDX (EGFR T790M) cell line was obtained from Dr. Rishi’s Laboratory at Wayne State University and Lung CAFs (AA0022) was obtained from Dr. Noyes Laboratory, Moffit Cancer Center. A total of 10 × 10^6^ cells were mixed homogeneously with the hydrogel using a spatula in 10:1 ratio and printed with INKREDIBLE bioprinter (Cellink, Sweden). For co-culture, 5 × 10^6^  cells of each PDX and CAFs were homogeneously mixed with the hydrogel and printed with print head 1 in INKREDIBLE bioprinter. 22 G needle (CELLINK, Sweden) was used for bio-printing throughout the experiment. After printing, scaffolds were crosslinked with 100mMCaCl_2_ crosslinker solution and transferred to 6 well plates. The printed cell laden scaffolds were cultured in DMEM/F12 and RPMI media supplemented with B27 supplement, recombinant human epidermal growth factor (EGF) and recombinant human basic fibroblast growth factor (bFGF).

### Cell viability

Printed cell viability was studied by using Live-Dead Assay Kit (Biotium Inc.) Briefly, printed cells were stained with 2uM calcein AM/4 uM EthD-III for 1 h at 37 °C, 5% CO_2_. Cell laden scaffolds were washed twice with serum free media and photographs were taken by using Olympus IX70 microscope. Percentage of live and dead cells were determined by counting the cells using Image-J (NIH, USA) software and a histogram was made to compare the cell viability in different printed groups.

### Microscopy for spheroid formation

Nucleus and Actin staining was performed to identify co-culture spheroid growth inside the printed scaffold. Briefly, cell laden scaffolds were fixed using 4% formaldehyde for 1 h at room temperature and washed twice with HBSS for 15 minutes each. The scaffolds were then permeabilized with 0.1% Triton X-100 in HBSS. NucBlue and ActinGreen488 (Life Technologies) staining was performed according to manufacturer protocol. Images were taken by using confocal microscope (Nikon).

### Immuno-fluorescence staining for cellular crosstalk

Immuno-fluorescence staining was performed to determine the cross-talk between printed PDX and CAF in co-culture spheroid. After 15 days, the co-culture spheroids were removed from the printed scaffold by dissolving the scaffold in Algimatrix dissolving buffer (Gibco). Co-culture spheroids were fixed with 4% formaldehyde for 1 h at room temperature and then treated with 0.1% triton X-100 for another 1 h at 37 °C. The co-culture spheroids were then washed in PBS twice and blocked overnight using 5% BSA in PBST. E-cadherin, vimentin and alpha-SMA antibodies with 1:100 dilutions in 3%BSA were incubated overnight. FITC and Texas Red conjugated secondary antibodies were used for 2 h and finally NucBlue staining was performed. Images were taken by using a fluorescence microscope (Olympus).

### Statistical analysis

The experimental results were expressed as means ± standard deviation. Statistical analyses were performed by Students T Test. The p value < 0.05 were considered as statistically significant.
