# Author Correction: Characterization and printability of Sodium alginate -Gelatin hydrogel for bioprinting NSCLC co-culture

**DOI:** 10.1038/s41598-020-58952-1

**Published:** 2020-01-29

**Authors:** Arindam Mondal, Aragaw Gebeyehu, Mariza Miranda, Divya Bahadur, Nilkumar Patel, Subhramanian Ramakrishnan, Arun K. Rishi, Mandip Singh

**Affiliations:** 1 College of Pharmacy and Pharmaceutical Sciences, Florida A&M University, Tallahassee, Florida 32307 USA; 2 Department of Chemical & Biomedical Engineering, FAMU-FSU College of Engineering, Florida State University, Tallahassee, FL 32310 USA; 3 John D. Dingell VA Medical Center, Detroit, MI 48201 USA; 4 Department of Oncology, Karmanos Cancer Institute, Wayne State University School of Medicine, Detroit, MI 48201 USA

Correction to: *Scientific Reports* 10.1038/s41598-019-55034-9, published online 27 December 2019

The Acknowledgements section in this Article is incomplete.

“This project was supported by NSF-CREST center for Complex Material Design for Multidimensional Additive processing (CoManD) award # 1735968.”

should read:

“This project was supported by NSF-CREST center for Complex Material Design for Multidimensional Additive processing (CoManD) award # 1735968, and FAMU center of Health disparities research grant, RCMI 5U54MD007582-35 from NIH.”

